# Comparative Study of Extracellular Vesicles from the Urine of Healthy Individuals and Prostate Cancer Patients

**DOI:** 10.1371/journal.pone.0157566

**Published:** 2016-06-15

**Authors:** Olga E. Bryzgunova, Marat M. Zaripov, Tatyana E. Skvortsova, Evgeny A. Lekchnov, Alina E. Grigor’eva, Ivan A. Zaporozhchenko, Evgeny S. Morozkin, Elena I. Ryabchikova, Yuri B. Yurchenko, Vladimir E. Voitsitskiy, Pavel P. Laktionov

**Affiliations:** 1 Laboratory of molecular medicine, Institute of Chemical Biology and Fundamental Medicine CD RAS, Novosibirsk, Russian Federation; 2 Dispensary department 2, Novosibirsk Regional Oncology Center, Novosibirsk, Russian Federation; 3 Group of microscopy, Institute of Chemical Biology and Fundamental Medicine CD RAS, Novosibirsk, Russian Federation; 4 Centre of Oncology and Radiotherapy, Novosibirsk Research Institute of Circulation Pathology Academician E.N. Meshalkin, Novosibirsk, Russian Federation; 5 Center of New Medical Technologies of ICBFM SB RAS, Novosibirsk, Russian Federation; Oxford Brookes University, UNITED KINGDOM

## Abstract

Recent studies suggest that extracellular vesicles may be the key to timely diagnosis and monitoring of genito-urological malignancies. In this study we investigated the composition and content of extracellular vesicles found in the urine of healthy donors and prostate cancer patients. Urine of 14 PCa patients and 20 healthy volunteers was clarified by low-speed centrifugation and total extracellular vesicles fraction was obtain by high-speed centrifugation. The exosome-enriched fraction was obtained by filtration of total extracellular vesicles through a 0.1 μm pore filter. Transmission electron microscopy showed that cell-free urine in both groups contained vesicles from 20 to 230 nm. Immunogold staining after ultrafiltration demonstrated that 95% and 90% of extracellular vesicles in healthy individuals and cancer patients, respectively, were exosomes. Protein, DNA and RNA concentrations as well as size distribution of extracellular vesicles in both fractions were analyzed. Only 75% of the total protein content of extracellular vesicles was associated with exosomes which amounted to 90–95% of all vesicles. Median DNA concentrations in total extracellular vesicles and exosome-enriched fractions were 18 pg/ml and 2.6 pg/ml urine, correspondingly. Urine extracellular vesicles carried a population of RNA molecules 25 nt to 200 nt in concentration of no more than 290 pg/ml of urine. Additionally, concentrations of miR-19b, miR-25, miR-125b, and miR-205 were quantified by qRT-PCR. MiRNAs were shown to be differently distributed between different fractions of extracellular vesicles. Detection of miR-19b versus miR-16 in total vesicles and exosome-enriched fractions achieved 100%/93% and 95%/79% specificity/sensitivity in distinguishing cancer patients from healthy individuals, respectively, demonstrating the diagnostic value of urine extracellular vesicles.

## Introduction

Prostate cancer (PCa) is the second most common cancer worldwide in males, with more than 1.1 million new cases diagnosed in 2012 (global cancer statistics, http://www.cancerresearchuk.org/). Despite five-year survival rate reaching 98% in developed countries, early PCa detection and accurate post-therapy monitoring for tumor recurrence, proliferation and metastatic potential is demanded. It can increase the quality of life for PCa patients, ensure timely diagnosis and survival of patients diagnosed at an advance stage. Despite a number of shortcomings and U.S. Preventive Services Task Force recommendation against its use, blood PSA is still used for PCa diagnostics [1]. Men with a high PSA are required to undergo additional tests such digital rectal exam or prostate biopsy, which are both uncomfortable and may cause adverse after-effects i.e. a needle biopsy can result in infection or prolonged bleeding afterwards. PCA3 assay despite very good initial performance [2,3] was later shown to possess low sensitivity and specificity (69 and 58%, correspondingly) [4]. Thus, a non-invasive test for PCa is still desired.

The prostate ejaculatory ducts empty directly into the urethra, carrying the prostate secretions into the urinary tract. Thus, urine represents a potentially valuable source of diagnostic material for monitoring the prostate. Indeed, it has been shown that cell-free DNA from the urine can be used for PCa diagnostics, and simple procedures like prostate massage can increase the amount of tumor-specific nucleic acids in urine and subsequently the efficacy of PCa diagnostics [5,6].

The low concentration of tumor-specific molecules demands a special protocol for their isolation from large urine volumes as well as a highly sensitive quantification assay. This seemingly decreases the attractiveness of urine as a source of diagnostic material. Recently, however, certain types of extracellular vesicles (EVs), enriched in biopolymers originating from cancer cells were found in urine [7–9]. The most interesting are exosomes, a subclass of extracellular vesicles ~ 30–150 nm in diameter, containing a portion of the parent cell cytoplasm [10]. Exosomes are released into the extracellular space after merging of multivesicular bodies with the cell membrane and are subsequently passed into the blood, urine and other biological fluids. In contrast, microvesicles are formed from the plasma membrane, and are more heterogeneous in size [11,12]. Both microvesicles and exosomes have been shown to contain a snapshot of the nucleic acid content of the parent cell [13].

A comprehensive analysis of the protein content of EVs found in urine showed the presence of proteins/transporters specific to cells of the kidney and urogenital tract [14,15]. Later, it was shown that sufficiently stable urine microvesicles carry miRNA, and also have small amounts of DNA at their surface [13] and, similar to blood EVs, have the potential to be used as a source of biomarkers for the detection of genitourinary pathologies [16].

There are examples of transcriptomics and proteomics studies of urinary EVs. Royo and colleagues performed transcriptomic profiling of urinary EVs obtained from prostate cancer and benign prostate hyperplasia patients using HumanHT-12 v4 Expression BeadChip and found two RNA transcripts, Cadherin 3, type 1 (CDH3) and CKLF-Like MARVEL Transmembrane Domain Containing 3 (CMTM3), exhibited the predicted behavior [17]. Overbye and colleagues reported a mass spectroscopy proteomic study of urinary exosomes in order to identify proteins differentially expressed in PCa patients and healthy male controls [18].

In the separate studies urine EVs were studied in respect to their size [19,20], protein, RNA, DNA and miRNA content [21,22]. Differences in EV isolation including different centrifugation regimes and filtration as well as use of different size measurement methodologies lead to the discrepancy in the results concealing diagnostic significance of different subclasses of EVs. It is still not clearly established what particles are enriched in protein or nucleic acids, and if there are morphological differences in EVs in health and disease.

Here we investigated EVs from cell-free urine obtained by different centrifugation and filtration protocols. Morphology, size distribution, protein, DNA and RNA content, as well as prostate cancer-specific miRNA markers were studied in cell-free urine fractions including total EV (TEV) and small EV (ERV) (below 100 nm). Special attention was given to the comparison of EVs from healthy individuals and prostate cancer patients and utility of EVs for prostate cancer diagnostics.

## Materials and Methods

### Study population and blood/urine collection

Blood (used only to determine the PSA level) and urine samples of 20 healthy male individuals (HD) and 14 previously untreated prostate cancer patients (PCa) were obtained from Center of New Medical technologies of ICBFM SB RAS and Regional Oncology Center (Novosibirsk, Russia) (Table 1). None of the patients had undergone surgical treatment or received chemotherapy prior to/at the time of sample collection.

**Table 1:** Overview of the study population.

	HD	PCa
	(n = 20)	(n = 14)
**Age**		
**Mean±SEM**	59±1.7	72±1.5
**Range**	48–73	63–82
**Total PSA, ng/ml**	1.1±0.15	20±3
**PCa stage**	N/A	T_2-3_N_X_M_X,1_
**T**_**2**_	N/A	7 (50%)
**T**_**3**_	N/A	7 (50%)
**N**_**X**_	N/A	14 (100%)
**M**_**X**_	N/A	13 (93%)
**M**_**1**_	N/A	1 (7%)
**Gleason score 5–6**	N/A	1 (7%)
**Gleason score 6**	N/A	5 (36%)
**Gleason score 7**	N/A	8 (57%)

The work was conducted in compliance with the principles of voluntariness and confidentiality in accordance with the “Fundamentals of Legislation on Health Care”. The study was approved by the ethics committees of ICBFM SB RAS and Novosibirsk Regional Oncology Center and written informed consent was provided by all participants.

### Urine fractionation and isolation of extracellular vesicles

To pellet cells, fresh urine was clarified by centrifugation at 400g, 20°C, for 20 min within 3 hours after collection. Supernatants were then centrifuged at 17000g, 20°C, for 20 min. Aliquots from both supernatants were immediately frozen and stored at -20°C until use. Aliquots were thawed once.

Total EVs (TEV) were precipitated from the 17 000g supernatant by high-speed centrifugation at 100000g, 18°C, for 90 min, the pellet was washed with 10 ml PBS and re-suspended in 100–300 μl of PBS. To obtain the exosome-enriched fraction of vesicles (ERV) TEV fraction was size selected after first centrifugation by syringe filtration through a 0.1 μm pore filter (Minisort High-Flow, Sartorius) and precipitated by high speed centrifugation. The pellets were resuspended in 100–300 μl of PBS and immediately used for transmission electron microscopy observations. Preparations of EVs in PBS were snap frozen in liquid nitrogen and stored at -80°C until use. Aliquots were thawed once immediately before use. Where noted concentrations of the biopolymers were normalized to the initial urine volume according to dilution ratios calculated for each faction.

### Transmission electron microscopy

Fresh samples of extracellular vesicles (20 μl) were adsorbed for 1 min on copper grids covered with formvar film and stabilized by carbon. The grids were exposed for 5–10 sec on a drop of 0.5% uranyl acetate, then the excess of fluid was removed using filter paper and the grids were air dried. The size distribution of vesicles was evaluated using 36 TEM images from three healthy donors and three PCa patients (six images per patient, a median of ~39 vesicles per image, no less than 200 per patient). Counting was performed manually, with increments of 10 nm; for example, the 31–40 nm group contains vesicles sized ≥30.1 nm, but ≤40 nm. To evaluate the expression of surface marker proteins, 10 μl of thawed suspension was incubated for 18 h on an orbital shaker with 10 μl of 0.5% BSA/PB (phosphate buffer) and 3 μl of mouse monoclonal anti-CD63, CD24 and CD9 antibodies (Abcam, 100 μg/ml). After incubation, vesicles were adsorbed for 1 min on copper grids covered with formvar film and stabilized by carbon, rinsed twice with PBS, and incubated with a protein A-colloidal gold conjugate for 2 h in a humid chamber on a shaker at room temperature followed by two washes with PBS, then stained with phosphotungstic acid, as described previously [23,24]. Grids were analyzed using a JEM 1400 (80 kV, Jeol, Japan) transmission electron microscope supplied with a digital camera Veleta (Olympus SIS, Germany). The measurements were performed using iTEM (Olympus SIS, Germany) software.

### Protein measurements

Protein concentrations were measured using the NanoOrange Protein Quantitation Kit (Invitrogen, USA) according to the manufacturer's protocol. Preparations of EVs were lysed for 10 min on ice in lysis buffer (125 mM Tris-HCl, pH 6.8, 4% SDS, 0.1 M DTT), incubated at 95°C for 10 min with a working solution of NanoOrange reagent. Fluorescence was measured on a VersaFluor^™^ fluorometer at 480 nm excitation and 580 nm emission filters against standard solutions of BSA.

### Nucleic acid isolation and analysis

Total DNA and RNA from the total microvesicles and exosomes were isolated using a DNA or RNA isolation kit (Biosilica Ltd, Russia), followed by precipitation of the nucleic acids using acetone and triethylamine, as described earlier [25]. DNA concentration was measured using multiplex TaqMan PCR (α-satellite elements and LINE1 repeats) [25]. RNA samples were treated with DNase I (Fermentas) at 37°C for 30 min, and one RNA sample from a PCa patient was further treated with RNase A (Fermentas). The RNA size distribution was analyzed using an Agilent 2100 Bioanalyzer capillary electrophoresis system and RNA 6000 Pico Kit.

MiRNA from all fractions were isolated using single-phase protocol as described [26]. Concentrations of miRNA (miR-16, miR-19b, miR-25, miR-125b, and miR-205) were determined by TaqMan PCR after reverse transcription with stem-loop primers, as described earlier [26]. Primers and probes for reverse transcription and quantitative PCR are listed in S1 Table.

### Statistical analysis

Statistical analyses were performed using GraphPad Prism 5 software. The specificity and sensitivity of the analytical systems was characterized by Receiving Operator Characteristic curves (ROC-curves). Threshold cycle values (Ct) values for all miRNAs obtained in qRT-PCR were normalized to miR-16 to obtain delta Ct values (dCt). Normalizing was done by subtracting Ct values for miR-16 from Ct values for target miRNA for each sample.

## Results

The isolation protocol used in this study yielded four fractions. First (400g supernatant) fraction is clarified cell-free urine and presumably contains all types of vesicles present in the urine as well as urine proteins, protein aggregates, cell fragments and debris, and cell-free nucleic acids not associated with membrane vesicles. Second fraction (17 000g supernatant) contains urine EVs, soluble urine protein and cell-free nucleic acids not packed in EVs. Third fraction containing total extracellular vesicles (TEV) is a pellet of high-speed centrifugation and is comprised of exosomes and smaller microvesicles (up to 200–300 nm). Finally, the exosome-rich fraction of vesicles (ERV) obtained by 0.1 μm filtration of TEV fraction is enriched in smaller vesicles, including exosomes. The composition of EVs was studied in TEV and ERV fractions, except miRNA concentration, which was measured in all fractions.

### Description of urine extracellular vesicles

**Fig 1:**
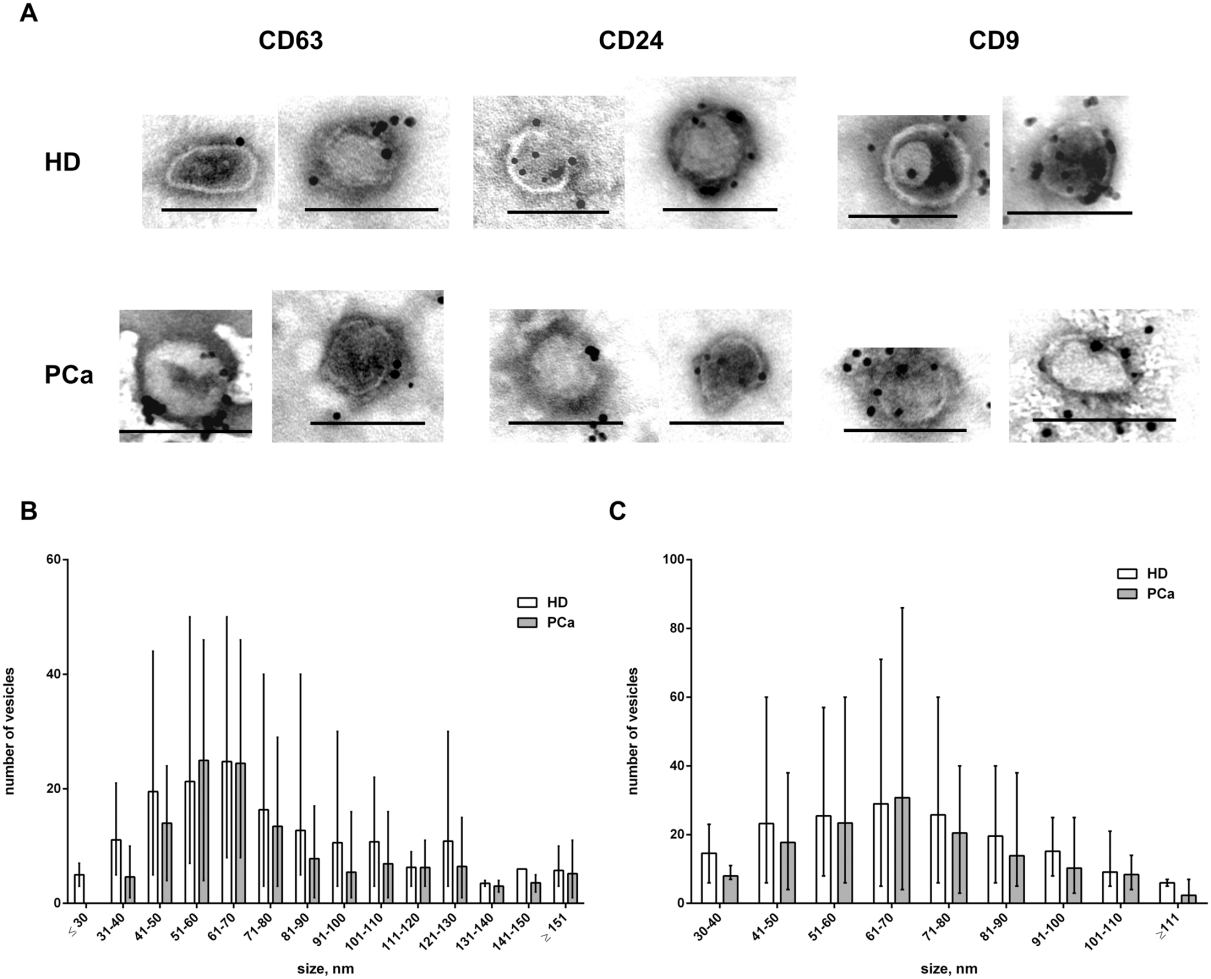
Appearance and size of urine EVs. (A) TEM images of EVs from urine of HD and PCa patients before and after 0.1 μm filtration. (B) Size distribution of EVs before and after 0.1 μm filtration. Mean size with error bars for range. Additional TEM images can be found in S1 Fig.

According to TEM, TEV from urine of healthy donors and PCa patients contained EVs sized from 20 to 230 nm (Fig 1A). The majority of vesicles had the appearance of spherical bubbles or “cups” hinting at their exosomal/endocytic origin. Vesicles ranging 30–100 nm (exosome size range) amounted to 95% and 90% of the total number of vesicles in TEV samples from HD and PCa respectively; this difference was not significant (Mann-Whitney test) (Fig 1B).

Filtration of TEV fraction through a 0.1 μm filter increased the portion of 30–100 nm exosome-like vesicles up to almost 100%. The vesicles exceeding 110 nm were almost completely eliminated by 0.1 μm filtration (Fig 1B). Immunogold staining of 0.1 μm filtered samples with antibodies against CD63, CD9 and CD24 was positive for virtually all 30 to 100 nm vesicles, suggesting their exosomal origin (Fig 1A).

### Quantification of total protein in urine EVs

Indirectly, the number of microvesicles and exosomes can be estimated from the total protein concentration in their preparations. In our study protein concentration in preparations of urine EVs was measured by NanoOrange Protein Quantification Kit, which includes heating of sample with detergent to lyse the vesicles.

**Fig 2:**
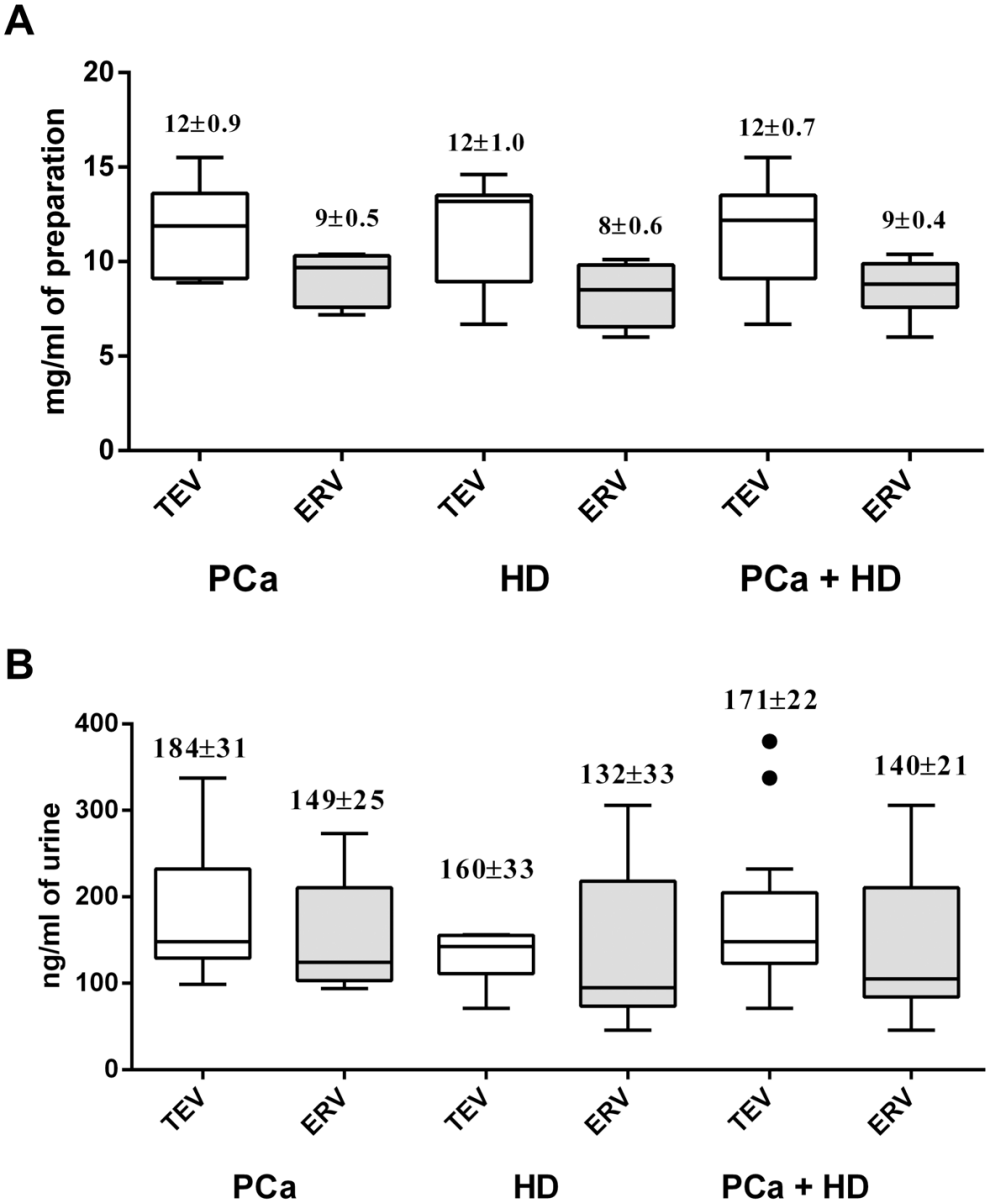
Protein concentration in urine EVs. Tukey box plots of concentration of total protein in preparations of EVs isolated from the urine of PCa patients and HD were determined using NanoOrange fluorescent dye and presented as mg per ml of probe (A) or ng per ml of urine (B).

The protein concentration in the preparations of urine EVs did not differ between cancer patients and healthy individuals (Fig 2). The concentration of protein in preparations of urine EVs was 12±0.7 mg/ml and 9±0.4 mg/ml, respectively (Fig 2A), which corresponds to 171±22 ng and 140±21 ng per ml of urine used (Fig 2B). Considering the normal concentration of urine protein (about 33 μg /ml) and the dilution during the EV isolation including resuspension and centrifugation steps, EV preparations contained no more than 3 ng/ml of remaining urine protein. Thus protein content in preparations of EVs amounts to approximately 0.6% of total urine protein. It should be noted that only 75% of the total protein of the TEV was associated with ERV, which constitute 90–95% of all vesicles. Thus, 5–10% of large vesicles (>110 nm) contained 25% of the total protein associated with EV, demonstrating the different nature of small (100<nm) and large protein-rich EV.

### Total DNA and RNA content of urine EVs

**Fig 3:**
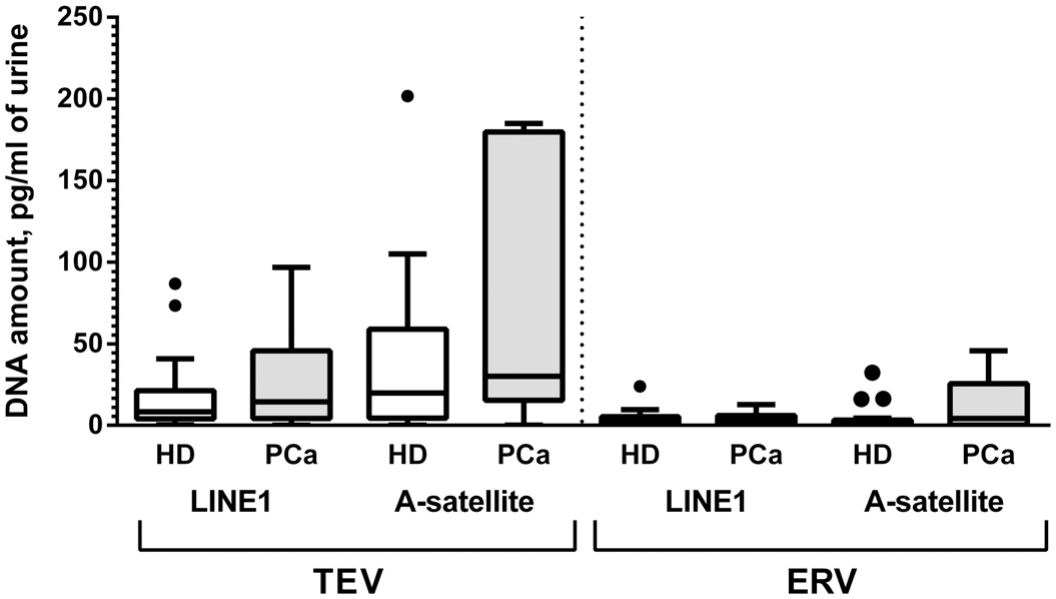
DNA in preparations of urine EVs. Tukey box plots of DNA concentration in preparations of EVs isolated from the urine of PCa patients and HD was determined using quantitative multiplex TaqMan PCR for LINE1 and α-satellite human DNA repeats.

The concentration of extracellular DNA did not differ in the preparations of TEV and ERV isolated from the urine of PCa patients and HD; median DNA concentration in TEV was 18 pg/ml (maximum value 200 pg/ml) and median DNA concentration in ERV fraction was 2.6 pg/ml (maximum value 50 pg/ml) (Fig 3).

**Fig 4:**
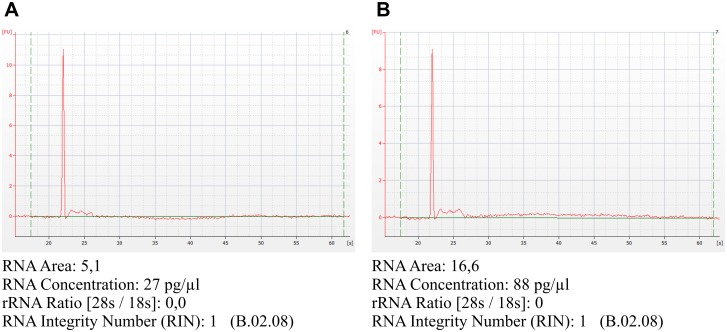
Total RNA in preparations of urine EVs. Bioanalyzer traces of total RNA from ERV fraction of the urine of HD (A) and PCa patient (B). The data from Agilent 2100 Bioanalyzer with 25 nt RNA fragment as an internal standard.

RNA content in EVs from healthy donors and PCa patients, both in TEV and ERV fractions, was comprised of 30–180 nt molecules, as demonstrated using an Agilent 2100 Bioanalyzer and RNAse treatment. The obtained profiles were close to those demonstrated by Royo et al. [17] and typical for exosomal RNA. Total RNA concentration was no more than 290 pg/ml of urine (Fig 4).

### Extracellular miRNA from urine EVs

**Fig 5:**
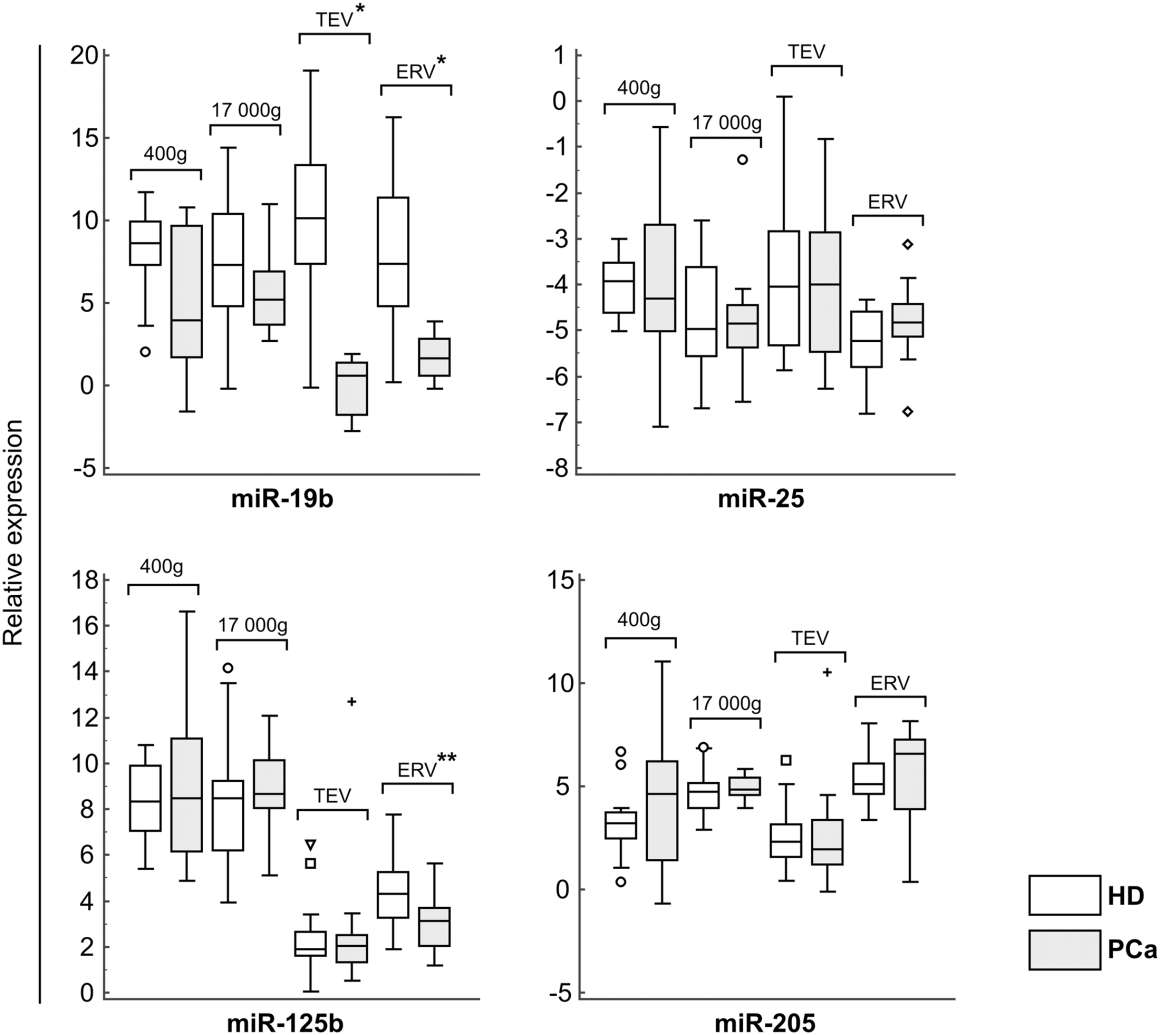
Relative miRNA expression in urine EVs. Tukey box plots of miRNA expression in all fractions of urine. Asterisks (*) indicate statistical significance. *P<0.0001; **P = 0.0150.

To select miRNAs potentially overexpressed in PCa patients the available data of the expression of miRNA in PCa were analyzed [27,28]. Four miRNAs (miR-19b, miR-25, miR-205, and miR-125b) were selected as potential markers of PCa. The control miRNA (miR-16) was shown earlier to be relatively stably expressed and used in the current study to normalize expression level of target miRNAs. The concentrations of 5 miRNAs were measured in the 400g and 17000g supernatants, TEV and ERV fractions by RT TaqMan PCR (Fig 5).

Using the Mann-Whitney test, significant differences between the dCt values of miR-19b in TEV and ERV fractions (P<0.0001), and the dCt of miR-125b in ERV (P = 0.0150) from HD and PCa patients (Table 2) were found. ROC analysis of miRNA expression in all fractions demonstrated that only the difference in the concentration of miR-19b in EV was diagnostically significant. It allowed differentiating of PCa patients from clinically healthy individuals with 100% specificity, 93% sensitivity and 95% specificity, 79% sensitivity, for urine TEV and ERV fractions, correspondingly (Table 2). Thus, we demonstrated that the quantification of miRNAs (particularly miR-19b) in urine microvesicles can be used as a primary or at least an auxiliary criterion for the diagnosis of prostate cancer.

**Table 2:** Expression and diagnostic value of miRNA in HD and PCa patients. Analytical performance ROC analysis and statistical analysis of the results (non-parametric Mann-Whitney test) of miRNA expression in urine.

	Fraction			
	400g	17000g	TEV	ERV
**miR-205**				
ddCt, HD vs PCa	-1,03	-0,40	-0,81	-0,25
Sensitivity	64%	71%	64%	57%
Specificity	87%	55%	42%	75%
P value	0.2658	0.1324	0.9855	0.6618
**miR-125b**				
ddCt, HD vs PCa	-0,97	-0,35	-0,38	1,26
Sensitivity	50%	71%	64%	86%
Specificity	67%	65%	53%	65%
P value	0.7107	0.2142	0.9564	**0.0150**
**miR-25**				
ddCt, HD vs PCa	-0,01	0,11	0,39	-0,46
Sensitivity	43%	64%	50%	71%
Specificity	80%	55%	53%	60%
P value	0.8272	0.5403	0.5848	0.1369
**miR-19b**				
ddCt, HD vs PCa	2,94	0,70	11,01	6,08
Sensitivity	50%	50%	**93%**	**79%**
Specificity	87%	60%	**100%**	**95%**
P value	0.6784	0.6119	**<0.0001**	**<0.0001**

**Fig 6:**
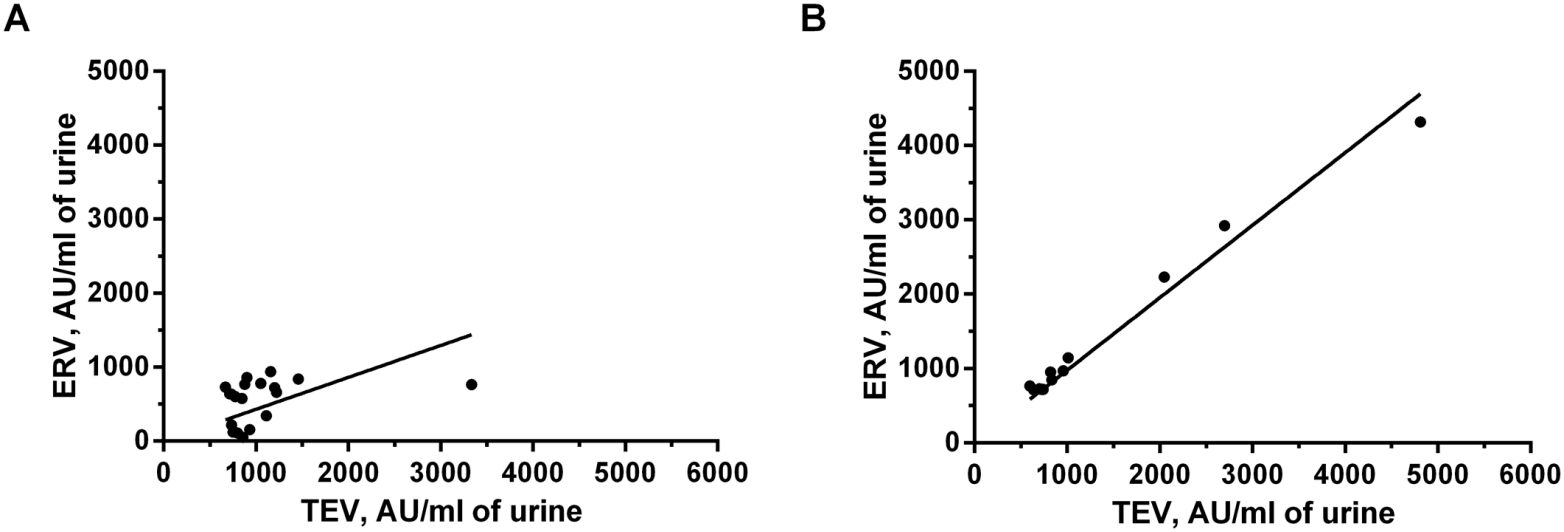
Example miR-19b correlation plots. Correlation plots of miR-19b concentration in TEV and ERV fractions of healthy individuals (A) and prostate cancer patients (B). AU—arbitrary units of concentration.

To look at the distribution of miRNA between the fractions R coefficients for correlations between the concentrations of the miRNAs recalculated to the starting urine volume, taking into account all dilutions and assuming equal efficacy of RT and PCR were calculated (Table 3). Meaningful correlations (R>0.6) between miRNA concentrations in ERV and TEV were discovered for miR-16 and -205, both in both groups, for miR-19b in PCa patients and miR-25 in HD. The miR-25 concentration was inversely correlated between TEV and the 17 000g supernatant in PCa patients, while the miR-205 concentration demonstrated a slight correlation between ERV and the 400g supernatant. The data presented in Table 3 demonstrated that miRNAs are present in both EVs and in the EV-free fraction. For example, the miR-16 concentration demonstrated a noticeable correlation between the 400g supernatant and TEV in healthy donors (R = 0.58, P = 0.009) and was also highly correlated between urine TEV and ERV in both healthy donors (R = 0.9, P<0.001) and PCa patients (R = 0.89, P<0.001). These data indicate that miR-16 is predominantly located in urine exosomes regardless of health status. As for miR-19b, in PCa patients but not in healthy individuals it was preferentially found in the urine ERV fraction (Fig 6). Redistribution between EVs and vesicle-free fraction of urine presumably occurs during the development of prostate disease, as suggested by the absence of any correlation in healthy donors. Definite redistribution between factions of urine was also demonstrated for miR-25, which was detected in urine ERV/TEV in healthy donors (R = 0.74, P = 0.0003), but in PCa patients it was found in the 17000g vesicle-free fraction (R = -0.64, P = 0.015, 17000g/microvesicles). The two remaining miRNAs, miR-205 and miR-125b, were mainly found in TEV in both healthy donors and PCa patients.

**Table 3:** Spearman’s correlation of miRNA expression between the fractions. Concentration was normalized to the starting urine volume.

	HD			PCa		
	17000g	TEV	ERV	17000g	TEV	ERV
**miR-16**						
**400g**	0.08 (P = 0.7)	**0.58 (P = 0.009)**	0.43 (P = 0.058)	0.009 (P = 0.98)	-0.03 (P = 0.91)	-0.09 (P = 0.75)
**17000g**		0.12 (P = 0.62)	0.06 (P = 0.8)		0.03 (P = 0.93)	0.2 (P = 0.49)
**TEV**			**0.9 (P<0.00001)**			**0.89 (P = 0.00002)**
**miR-19b**						
**400g**	-0.35 (P = 0.12)	0.16 (P = 0.52)	-0.006 (P = 0.98)	0.22 (P = 0.45)	0.32 (P = 0.27)	0.37 (P = 0.19)
**17000g**		0.16 (P = 0.5)	0.12 (P = 0.6)		0.33 (P = 0.25)	0.32 (P = 0.27)
**TEV**			0.42 (P = 0.07)			**0.89 (P = 0.00002)**
**miR-25**						
**400g**	0.27 (P = 0.25)	0.43 (P = 0.06)	0.26 (P = 0.27)	-0.51 (P = 0.06)	0.13 (P = 0.65)	0.39 (P = 0.17)
**17000g**		-0.02 (P = 0.94)	-0.29 (P = 0.21)		**-0.64 (P = 0.015)**	-0.39 (P = 0.17)
**TEV**			**0.74 (P = 0.0003)**			0.46 (P = 0.09)
**miR-205**						
**400g**	-0.04 (P = 0.86)	**0.53 (P = 0.02)**	**0.57 (P = 0.009)**	0.42 (P = 0.14)	0.43 (P = 0.12)	0.43 (P = 0.13)
**17000g**		0.21 (P = 0.4)	0.02 (P = 0.95)		0.43 (P = 0.13)	0.15 (P = 0.6)
**TEV**			**0.9 (P<0.00001)**			**0.74 (P = 0.002)**
**miR-125b**						
**400g**	0.25 (P = 0.29)	0.25 (P = 0.3)	0.31 (P = 0.18)	-0.19 (P = 0.52)	0.28 (P = 0.34)	0.28 (P = 0.33)
**17000g**		0.32 (P = 0.18)	0.37 (P = 0.11)		-0.28 (P = 0.34)	-0.35 (P = 0.22)
**TEV**			**0.95 (P<0.00001)**			**0.95 (P<0.00001)**

This shows that miR-19b was redistributed in PCa patients and was found mainly in the EV in contrast to HD, where is appeared in cell-free and vesicle-free form. This suggests active secretion of miR-19b containing vesicles by tumor cells. Thus, distribution of particular miRNAs between vesicle-free complexes and different types of EVs can be an additional diagnostic criterion along with the changes in miRNA expression and must be taken into consideration in the development urine miRNA-based diagnostics.

## Discussion

Distinct populations of extracellular vesicles including exosomes (30–100 nm), prostasomes (50–500 nm), estosomes (50–1000 nm), oncosomes (50–500 nm) and microvesicles (100–1000 nm) are found in prostatic fluid and urine [8]. Urine exosomes are relatively small vesicles with a spherical [29], saucer [30] or cup shape [31,32] that contain proteins, mRNAs and microRNAs (miRNA) [33] and are released by cells in all segments of the nephron and the urogenital tract [34]. Exosomes produced by prostate cells travel with the prostate secretions via prostate ejaculatory ducts that empty directly into the urethra and pass into the urine where they can be readily detected [35].

A combination of size, morphology and presence of specific surface markers is commonly used to identify extracellular vesicles as exosomes. Antibodies to the tetraspanin receptors CD63 and CD9, which mediate the adhesion of exosomes to the recipient cell surface [36,37], and CD24, a marker of actively dividing and differentiating cells [38,39] are frequently found in exosomes. We have demonstrated that cell-free urine of healthy donors and PCa patients contains EV sized from 20 to 230 nm; no less than 90% of these vesicles fell into the 30–100 nm range of, with the abundance of 40–80 nm vesicles. The majority of vesicles below 100 nm were CD63, CD24 and CD9 positive and, therefore, they can be considered to be exosomes. These findings in general correlate with other reports demonstrating that the size of urine exosomes varies from 30 to 100 nm [7,22,33,34,40] and majority of them fall into the 35–40 nm range [14,41]. Despite the filtration of the EVs through 0.22 μm Royo et al [19] reported a significant number of 300–400 nm vesicles, along with the abundance of 100–200 nm vesicles as measured by NanoSight LM10 (Malvern, UK). Other authors found particles of similar size (149 ± 20 nm) in the urine of PCa patients and HD by dynamic light scattering using Zetasizer Nano ZS (Malvern Instruments Ltd, Worcestershire, UK). Overestimation of the particle size by NanoSight LM10 and Zetasizer Nano ZS, as well underestimation of size by TEM [42], combined with the effect of freeze/thaw cycle on EVs size could potentially interfere with the EV size distributions and comparisons of EV profiles between studies. In our study TEM images before and after 0.1 μm filtration demonstrate minor influence of TEM sample preparation on EV size.

We have also estimated the protein content of urine extracellular vesicles. It has been previously reported that 48% of the total urinary protein is contained in sediments, 49% is soluble [43] and 3% is contained in exosomes [16,44], which carry both common [45] and unique sets of proteins [46]. The spectrum of proteins found in urine exosomes includes more than three thousand entries [47] and continues to expand rapidly [45]. Some authors report a concentration of protein in urine exosomes from healthy donors ranging from 2.78 to 11.8 μg/ml of urine [29,44]; other results vary from 0.11 to 0.635 μg/ml of urine [48]. Overbye and colleagues studied the exosome proteins from urine of cancer patients and healthy controls. Protein concentration was measured by BCA assay kit based on colorimetric detection and quantitation of total protein. This method yielded 0.20 ± 0.11 (*n* = 15) μg exosomal protein/ml urine [18]. According to our data, the protein concentration in TEV and ERV fractions was 171±22 ng/ml and 140±21 ng/ml, respectively, and did not differ significantly between healthy donors and PCa patients. It should be noted, that NanoOrange provides high specificity of protein detection and the protocols used for the isolation of urine EVs in this study included multiple washes and re-precipitation steps, decreasing the contamination of EV preparations with urine protein. Taking into consideration the size distributions of ERV and larger vesicles (Fig 1), a trivial evaluation of their inner volume demonstrates that exosomes and larger vesicles have an approximately equal inner volume. As larger vesicles carry approximately 25% of total EV protein, it is logical to assume that biopolymer cargo of small vesicles in ERV fraction and larger vesicles is vastly different.

Furthermore, we showed the presence of DNA, RNA and miRNA in urine EVs, including exosomes, which can be a convenient source of material for the diagnosis of diseases of the urogenital tract, including malignancies of the prostate, kidney and bladder. The concentration of EV-associated DNA was low (picograms per milliliter of urine) and does not suggest any diagnostic implication. Allegedly, this DNA may be associated with the surface of vesicles, similar to 25–1500 nt DNA fragments sensitive to DNase treatment found in in preparations of urine exosomes by Miranda et al. [13]. Double-stranded genomic DNA was also previously found in preparations of serum exosomes [49] and exosomes released from pancreatic cancer cells and GBM cell lines [49,50] in quantities comparable with those found in our study. Besides, mitochondrial DNA was found in exosomes from C2C12 myoblasts [51], glioblastoma cells and astrocytes [52]. The presence of cell-surface receptors on exosomes, including those mediating DNA binding, can provide a reasonable explanation for these findings [53].

The RNA concentration in the ERV and TEV fractions of the urine (Fig 4) in healthy donors and PCa patients was also in the picograms per milliliter of urine range. The majority of extracellular RNA from healthy donors and PCa patients, both in exosomes and in the total microvesicles, were 30–180 nt long, and the RNA concentration was no more than 290 pg/ml of urine. This suggests the presence of tRNAs (73–93 nt), 5.8 rRNA (~150 nt), snoRNA (10–20 nt), snRNA (60–300 nt), piRNAs (29–30 nt), miRNAs (20–25 nt), siRNA (21–25 bp) and fragmented RNAs [7] in EV preparations. Alternate reports on RNA in urine exosomes/microvesicles are available. The data vary from the presence of mainly intact 18S and 28S rRNA and RNA concentration in a range from 381 ± 47 ng/ml to 410 ± 28 ng/ml [13] down to 0.2–0.4 ng/ml RNA [7]; some groups have reported that 80% of exosome RNA is miRNA [54]. Other authors have demonstrated that exosomal miRNA concentrations obtained by different miRNA isolation kits were 15 ng/ml of urine and higher [22,41]. In the current study, we used a miRNA isolation protocol based on the use of a single-phase aqueous-organic mixture specifically tailored for the isolation of miRNA and providing high efficacy of small RNA extraction [26]. Obviously, the discrepancies in reports concerning the size and concentration of RNA arise due to differences in stability during isolation and the efficacy of isolation by different methods, thus highlighting the inconsistency of the data. From the practical use standpoint miRNAs are still considered lucrative targets for diagnostics and their concentrations in urine EVs in range of tens of pg per ml looks reasonable for reliable detection.

Several studies have used cell-free urine or exosomes to isolate mRNAs and miRNAs. Nilsson was able to detect two known prostate cancer biomarkers, PCA3 and *TMPRSS2-ERG*, in exosomes isolated from urine of prostate cancer patients, first showing their potential for prostate cancer diagnostics [55]. An obvious reason to use exosomes as a source of miRNA-biomarkers instead of whole urine are the technical difficulties of handling large volumes of liquid during RNA extraction. Yet another reason would be the difference of miRNA composition and content in different components of urine–cells, EVs and membrane-free complexes.

Most studies use the urine cellular sediment obtained after low-speed centrifugation for miRNA analysis [56,57]. However, it was shown that a large proportion of RNA in the cell pellets of urine is low quality and degraded [22]. This is not surprising, as there is a high presence of RNase activity in the kidneys, bladder, and urinary tract to maintain sterility and protect the excretory system from microbial infections [58].

Unlike miRNA profiles found from plasma, cell-free urine does not contain a high abundance of miRNA. In a study by Cheng using 2.5 ml of cell-free urine only 12 miRNAs were reliably detected [22]. In contrast, 1 ml of plasma or serum can contain more than 500 miRNAs [59]. The low abundance of the detected miRNAs in cell-free urine further supports the presence of high RNase activity in the bladder compared with serum [60].

The volume (20 ml) of urine required to isolate a sufficient number of exosomes is far greater than the volume of cell-free urine (2.5 ml) required to isolate circulating miRNA. However, from the small pellet of exosomes isolated by ultracentrifugation, 184 miRNAs were detected. Only 7 miRNAs of the 184 were common to the cell-free urine, indicating the selective packaging of miRNA into EVs or effective elimination of vesicle–free miRNA complexes from cell-free urine [22].

In this study we have also demonstrated a higher concentration of miRNAs in the TEV and ERV fractions, as compared to urine supernatants. We have discovered that certain miRNAs can re-distribute between vesicles and vesicle-free complexes. This re-distribution may occur as a result of changes in current health status, as shown for miR-19b and miR-205. These data demonstrate the equal importance of sample preparation step and the selection of diagnostically significant miRNA markers for the development of diagnostic systems. Clearly, the distribution of miRNAs should be taken into consideration along with the analytical characteristics of the assay and the diagnostic efficacy of the marker.

Irrespective of the RNA extraction method, quantification of miRNA demonstrated that intact exosomes are enriched in miRNAs compared with the cell pellet and cell-free component of urine. The abundance of miRNA in exosomes further supports their function as transport vesicles that safely shuttle genetic material that would otherwise be degraded by RNase activity enclosed and protected by membrane of exosomes. The notion that urinary exosomes contain a stable source of miRNA makes urine an appealing biological source for discovery and detection of biomarkers.

## Conclusion

The data on urine EV including their size, protein DNA and RNA content in HD and PCa patients are presented. Extracellular vesicles isolated from the urine of PCa patients were shown to be a valuable source of diagnostically significant miRNAs. An in-depth study of the miRNA cargo of EVs present in the urine of PCa patients and HD will allow selecting a suitable set of markers for reliable PCa diagnostics.

## Supporting Information

S1 Fig TEM images of urine EVs of HD and PCa patient before and after 0.1 μm filtration. Scale bar 500 nm.
(TIF)

S1 Table Sequences of primers and probes used for reverse transcription and TaqMan qPCR. (DOCX)
