# Supplementary figures and images for: Comparative Study of Extracellular Vesicles from the Urine of Healthy Individuals and Prostate Cancer Patients
Source: PLoS One. 2016 Jun 15;11(6):e0157566. doi: 10.1371/journal.pone.0157566 (PMC4909321; doi:10.1371/journal.pone.0157566)

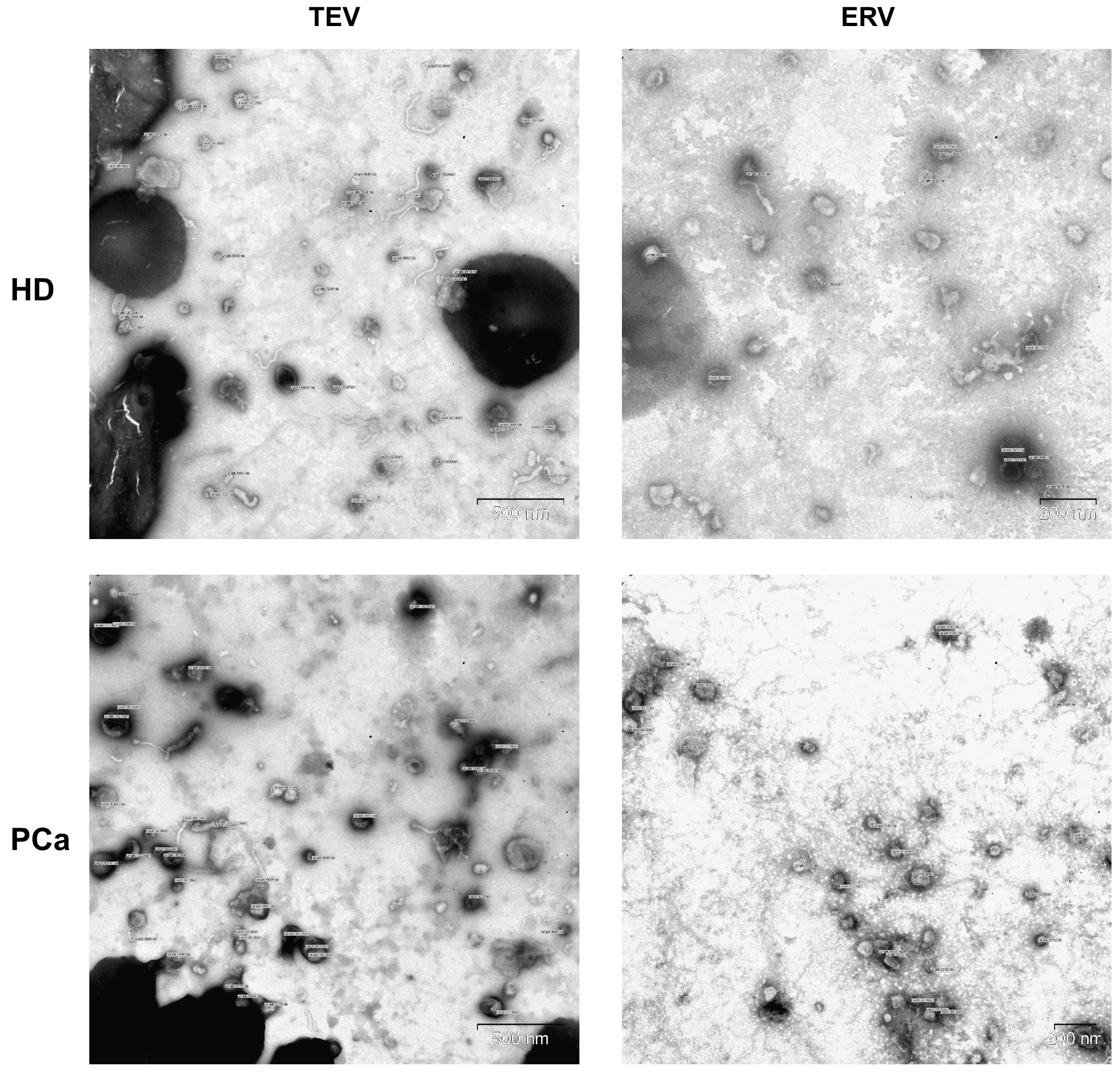

Supplement: S1 Fig — Scale bar 500 nm. (TIF) [file pone.0157566.s001.tif]
