# Supplementary material for: Comparative Study of Extracellular Vesicles from the Urine of Healthy Individuals and Prostate Cancer Patients
Source: PLoS One. 2016 Jun 15;11(6):e0157566. doi: 10.1371/journal.pone.0157566 (PMC4909321; doi:10.1371/journal.pone.0157566)
Supplement: S1 Table — (DOCX) [file pone.0157566.s002.docx]

**Supplementary materials**


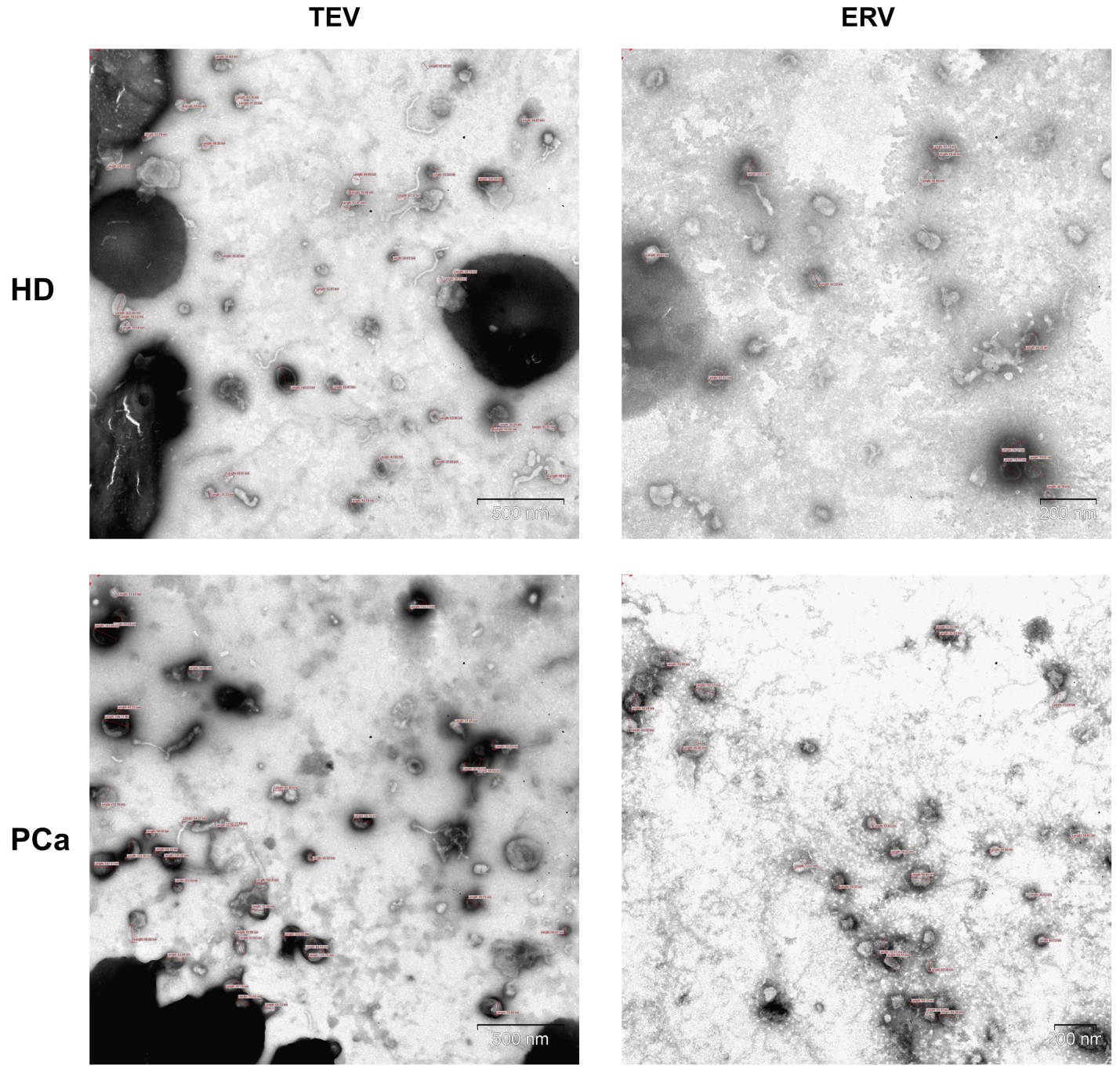


**Figure S1.** **TEM images of urine EVs of HD and PCa patient before and after 0.1 µm filtration.** Scale bar 500 nm.

**Table S1. Sequences of primers and probes used for reverse transcription and TaqMan qPCR.**

| **miRNA** | **Label** | **Sequence** |
| --- | --- | --- |
|  | **Universal Reverse Primer** | 5’-GTGCAGGGTCCGAGGT-3’ |
| **hsa-miR-16-5p** | **RT** | 5’-GTCGTATCCAGTGCAGGGTCCGAGGTATTCGCACTGGATACGACCGCCAA-3’ |
| **(miR-16)** | **Forward** | 5’-GCCCGTAGCAGCACGTAAATAT-3’ |
|  | **Probe** | 5’-(FAM)-GCACTGGATACGACCGCCAA-(FQ)-3’ |
| **hsa-miR-19b-3p** | **RT** | 5’-GTCGTATCCAGTGCAGGGTCCGAGGTATTCGCACTGGATACGACTCAGTT-3’ |
| **(miR-19b)** | **Forward** | 5’-CGCTGTGCAAATCCATGCAA-3’ |
|  | **Probe** | 5’-(FAM)-GCACTGGATACGACTCAGTT-(FQ)-3’ |
| **hsa-miR-205-5p** | **RT** | 5’-GTCGTATCCAGTGCAGGGTCCGAGGTATTCGCACTGGATACGACCAGACT-3’ |
| **(miR-205)** | **Forward** | 5’-CCTCCTTCATTCCACCGGA-3’ |
|  | **Probe** | 5’-(FAM)-GCACTGGATACGACCAGACT-(FQ)-3’ |
| **hsa-miR-25-3p** | **RT** | 5’-GTCGTATCCAGTGCAGGGTCCGAGGTATTCGCACTGGATACGACTCAGAC-3’ |
| **(miR-25)** | **Forward** | 5’-CCGCCATTGCACTTGTCTCG-3’ |
|  | **Probe** | 5’-(FAM)-GCACTGGATACGACTCAGAC-(FQ)-3’ |
| **hsa-miR-125b-5p** | **RT** | 5’-GTCGTATCCAGTGCAGGGTCCGAGGTATTCGCACTGGATACGACTCACAA-3’ |
| **(miR-125b)** | **Forward** | 5’-CGTCCCTGAGACCCTAACTT-3’ |
|  | **Probe** | 5’-(FAM)-GCACTGGATACGACTCACAA-(FQ)-3’ |
